# CFIm25 inhibits hepatocellular carcinoma metastasis by suppressing the p38 and JNK/c-Jun signaling pathways

**DOI:** 10.18632/oncotarget.24364

**Published:** 2018-01-31

**Authors:** Yunwu Wang, Yu Xu, Wei Yan, Ping Han, Jingmei Liu, Jin Gong, Dongxiao Li, Xiangming Ding, Han Wang, Zhuoying Lin, Dean Tian, Jiazhi Liao

**Affiliations:** ^1^ Department of Gastroenterology, Tongji Hospital of Tongji Medical College, Huazhong University of Science and Technology, Wuhan, Hubei Province, China

**Keywords:** hepatocellular carcinoma, CFIm25, alternative polyadenylation, EMT, metastasis

## Abstract

Alternative polyadenylation (APA), a post-transcriptional modification, has been implicated in many diseases, but especially in tumor proliferation. CFIm25, the 25 kDa subunit of human cleavage factor Im (CFIm), is a key factor in APA. We show that CFIm25 expression is reduced in human hepatocellular carcinoma (HCC), and its expression correlates with metastasis. Kaplan-Meier analysis indicated that CFIm25 is related to overall survival in HCC. Moreover, CFIm25 expression is negatively related to the metastatic potential of HCC cell lines. CFIm25 knockdown promotes cell invasion and migration *in vitro*, while overexpression of CFIm25 inhibits cell invasion and migration *in vitro* and inhibits intrahepatic and lung metastasis *in vivo*. Additional studies showed that CFIm25 disrupts epithelial-mesenchymal transition by increasing E-cadherin, that it inhibits HCC cell migration and invasion by blocking the p38 and JNK/c-Jun signaling pathways, and that CFIm25 knockdown increases the transcriptional activity of activating protein-1 (AP-1). These findings indicate that therapy directed at increasing CFIm25 expression is a potential HCC treatment.

## INTRODUCTION

Hepatocellular carcinoma (HCC) is the fifth most common cancer and the third leading cause of cancer-related death worldwide. It has a yearly fatality ratio of approximately 1, indicating that most patients do not survive one year [1]. HCC develops in cirrhotic patients in up to 90% of cases, mainly related to chronic viral hepatitis and alcohol abuse [2]. Invasion and metastasis are the underlying causes of poor long-term survival after clinical treatment in HCC [3]. HCC metastasis is a complex process that involves multiple factors, and its mechanism is not fully understood [4]. Therefore, in-depth research on the molecular mechanism of HCC metastasis might discover a potential intervention therapy.

Alternative polyadenylation (APA) is a fundamental molecular mechanism that influences the kinetics of gene regulation in diverse physiological and pathological states through the activity of messenger RNA (mRNA) 3′ untranslated regions (3′UTRs) [5, 6].Virtually 90% of human genes have distinct transcripts with variable 3′UTR lengths arising from multiple polyA sites, which is also the cause of transcriptome polymorphism [7–9].

Human cleavage factor Im (CFIm) is an essential component of the pre-mRNA 3′ processing complex that controls polyA site selection through the recognition of UGUA sequences upstream of the polyA site [10]. It contains three polypeptides of 25 kDa, 59 kDa, and 68 kDa that are designated CFIm25 (or CPSF5/NUDT21), CFIm59 (or CPSF7, cleavage and polyadenylation specificity factor 7), and CFIm68 (or CPSF6) [11]. Others have determined that the reduction of CFIm25 not only induces a global switch to the use of the polyA signal most proximal to the stop codon (pPAS) sites but also enhances cell proliferation [12, 13]. Studies have found that the expression of CFIm25 in tumor tissue is lower than in normal tissue in glioblastoma, and the level of CFIm25 is related to the proliferation of tumor cells [13, 14]. However, the function of CFIm25 in the occurrence and development of HCC remains unknown.

We show that CFIm25 inhibits cell invasion and metastasis of HCC though disruption of the epithelial-mesenchymal transition (EMT) process. These results support the hypothesis that CFIm25 prevents tumor invasion and metastasis in HCC. Moreover, we found that CFIm25 blocks the JNK and P38 signal pathways. Our findings indicate that CFIm25is a factor in HCC and is a candidate for directed therapy.

## RESULTS

### Downregulation of CFIm25 expression in HCC

**Figure 1:**
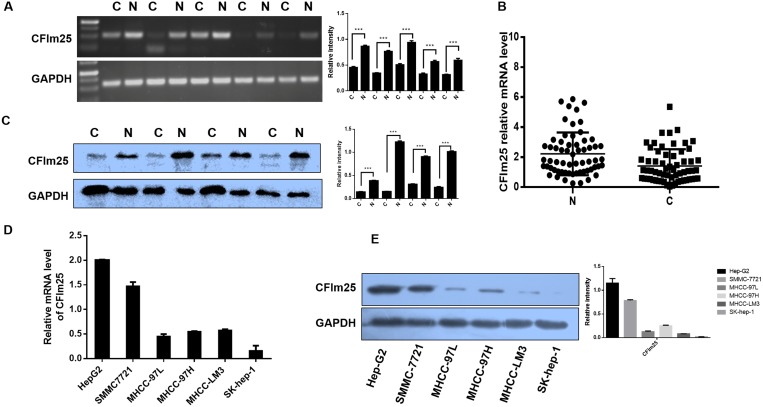
Downregulation of CFIm25 expression in HCC and metastatic HCC cells (**A**) PCR shows the expression of CFIm25 in HCC and the corresponding pericarcinoma tissue. N, liver pericarcinoma tissues; C, liver cancer tissues. (**B**) The expressions of CFIm25 in 62 pairs of HCC tissues and corresponding pericarcinoma tissues were measured by real-time RT-PCR. Data are represented as the mean ± SD. (**C**) Representative image shows CFIm25 expression in additional four pairs of HCC tissues and corresponding pericarcinoma tissues. N, liver pericarcinoma tissues; C, liver cancer tissues. (**D**) Real-time RT-PCR and (**E**) Western blot show the expression of CFIm25 in different HCC cell lines.

**Figure 2:**
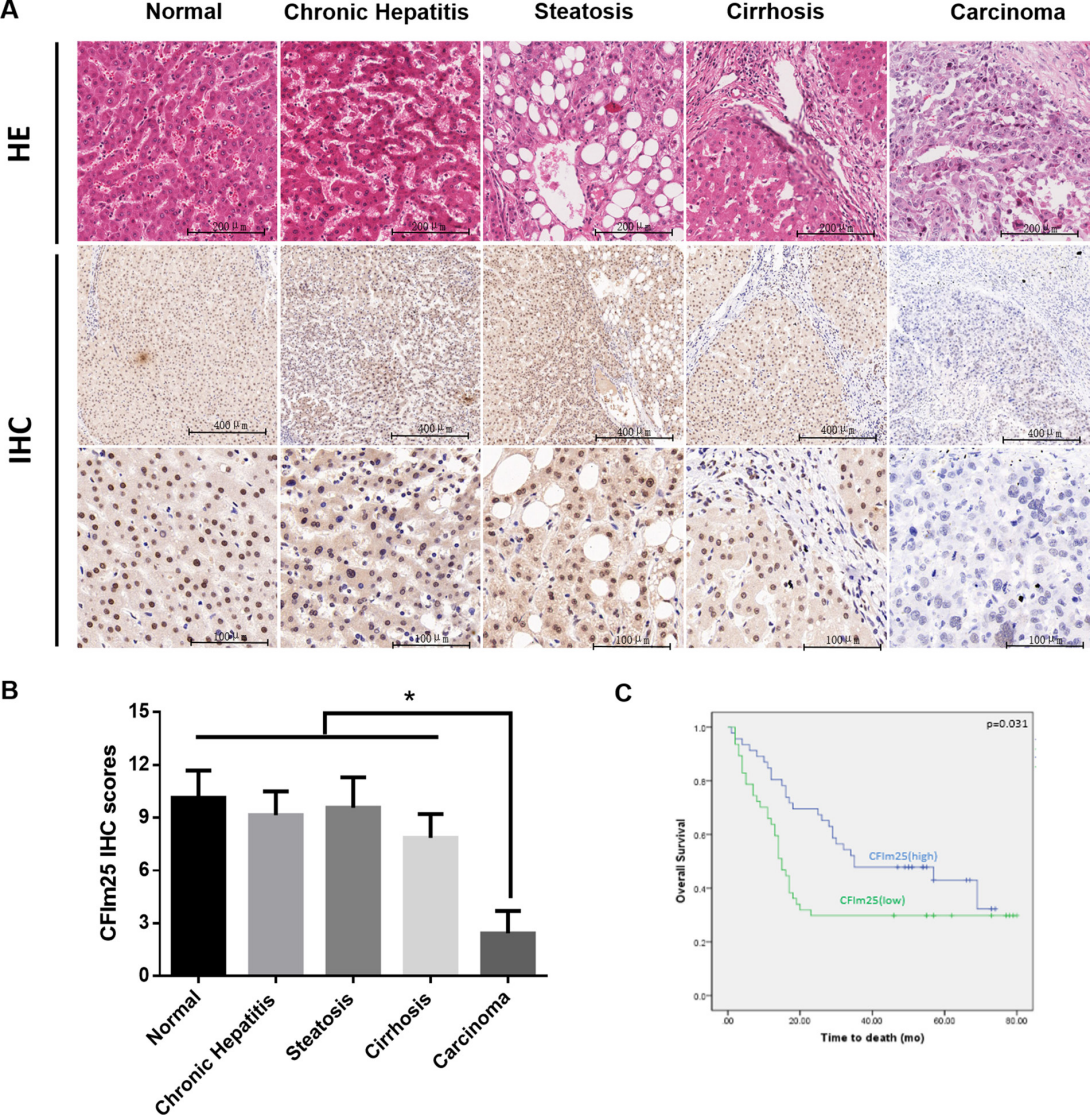
Combined analyses of CFIm25 in benign and malignant hepatic lesions (**A**) Representative images of CFIm25 expression in different hepatic lesions are shown (**B**) with semi-quantitative result displayed as mean ± SE. CFIm25 is present in the nucleus but not in the cytoplasm or on the membrane. (**C**) Kaplan-Meier survival curve for CFIm25. Median overall survival of the CFIm25-high and CFIm25-low cases was 44 and 31 months, respectively.

To determine the significance of CFIm25 expression in benign as well as malignant hepatic lesions, we analyzed CFIm25 levels in 122 HCC patients. We used RT-PCR and Western blotting to analyze the expression of CFIm25 in 62 pairs of tissue samples. Compared with the corresponding HCC tissue sample, CFIm25 was upregulated in pericarcinoma tissue samples (Figures 1A–1C). We applied immunohistochemistry to analyze CFIm25 protein levels in the other 60 HCC informative patients with various hepatic lesions, which mainly included cirrhosis, steatosis, chronic hepatitis, and cancer. CFIm25 protein was detected in the nuclei of hepatocytes, and representative images of immunohistochemical staining for noncancerous and cancerous tissue are shown in Figure 2. We found that CFIm25 in normal liver tissue, steatosis, and cirrhosis, expression was higher than in HCC.

To further investigate CFIm25 in HCC development, we studied a larger cohort of HCC patients. We recruited another 93 HCC patients with various tumor grades and stages. CFIm25 expression in tumor tissue was classified as high in 47 patients and low in 46 patients, basis for the immunohistochemistry score whose greater than or equal to 5 was defined as high expression, and less than 5 was defined as low expression. Expression of CFIm25 strongly correlated with the tumor progression. In comparing the clinicopathological parameters between groups, including age, sex, lymph node metastasis, tumor grading and TNM staging, we found the level of CFIm25 was significantly associated with lymph node metastasis (Person’s chi-square test, *P* = 0.011) and TNM staging (Person’s chi-square test, *P* = 0.029), whereas age, sex, and tumor grade showed no statistical significance with CFIm25 (Table 1). Median overall survival for the CFIm25-high and CFIm25-low cases were 44 and 31 months, respectively, indicating a significant difference in survival (Kaplan Meier log-rank test, *P* = 0.031; Figure 2C). Low levels of CFIm25 correlate with progression of human HCC and TNM stage correlates with tumor metastasis. Therefore, we investigated the CFIm25 expression in HCC cell lines with different metastatic potentials and found that the expression in Sk-hep-1, MHCC-LM3, and MHCC-97H was lower than in MHCC-97L, Hep-G2, and SMMC-7721 cells, which is contrary to the invasiveness of these cells [15, 16]. CFIm25 was frequently downregulated in HCC samples and metastatic HCC cells (Figure 1D, 1E).

**Table 1:** Correlations between CFIm25 and clinicopathological features of 93 HCC patients

Variables	CFIm25 staining		*P* value
	high expression( ≥ 5)	low expression( < 5)	
Age			0.105^a^
≤ 50	14	22	
> 50	32	25	
Gender			1.000^b^
Male	41	42	
Female	5	5	
Liver cirrhosis			0.350^a^
Absent	26	31	
Present	20	16	
Maximal tumor size			0.545^a^
≤ 5 cm	25	26	
> 5 cm	21	21	
Tumor location			0.139^a^
Left	4	7	
Right	16	23	
Others	26	17	
Pathological type			0.119^a^
Nodular type	24	20	
Giant mass type	14	10	
Others	8	17	
Lymph node metastasis			0.011a
Yes	10	22	
No	36	25	
Grading			0.238^a^
1~2	30	25	
3~4	16	22	
TNM staging			0.029^a^
I~II	29	19	
III~IV	17	28	

^a^Person Chi-square test;

^b^Continuity correction Chi-square test

### CFIm25 suppressed cell migration and invasion *in vitro*

**Figure 3:**
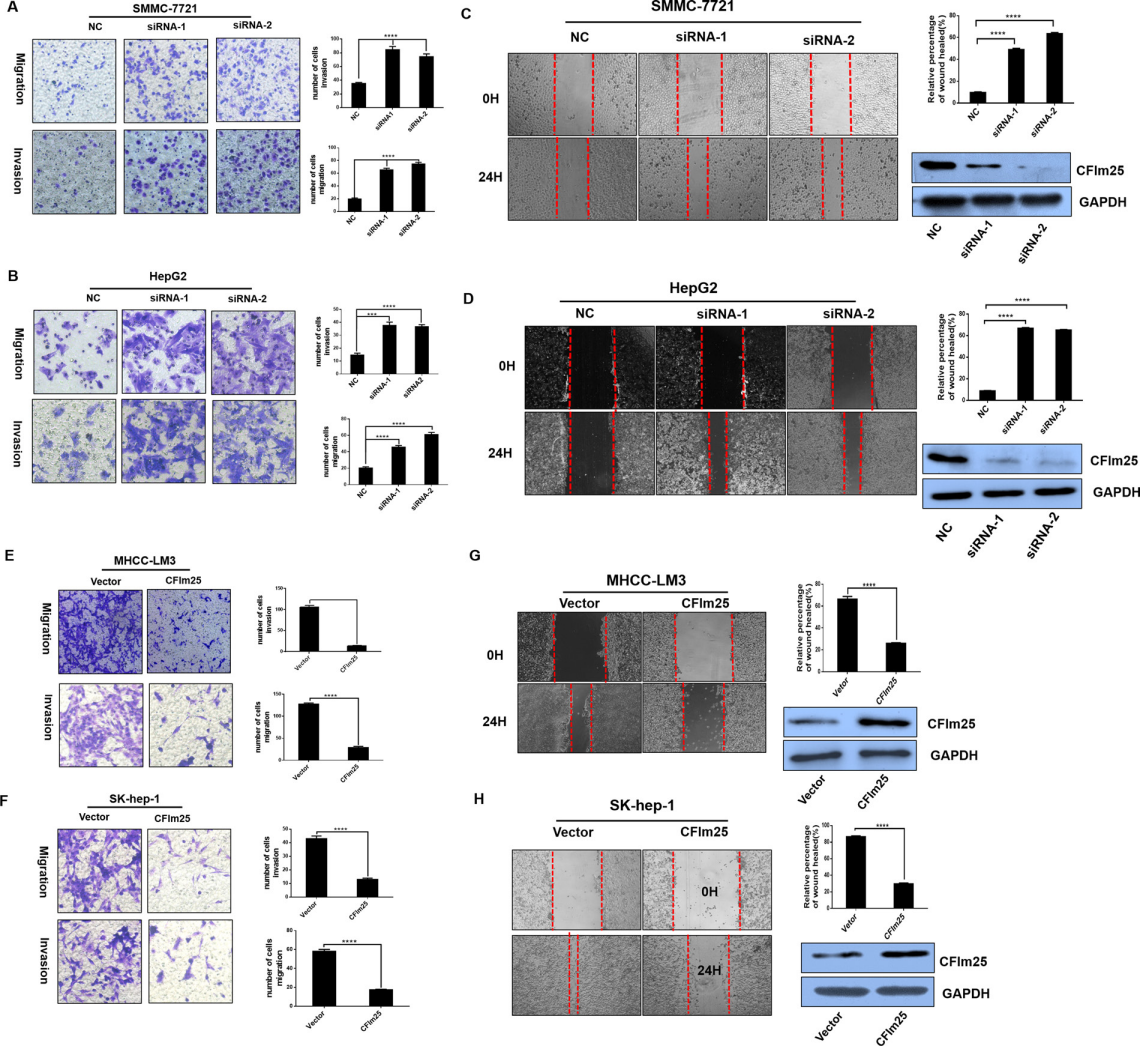
CFIm25 inhibits HCC cell migration and invasion *in vitro* CFIm25 knockdown promoted cell migration and invasion by transwell assay in (**A**) SMMC-7721 and (**B**) Hep-G2 cells. Overexpression of CFIm25 in (**E**) MHCC-LM3 and (**F**) Sk-hep-1 cells inhibits cell migration and invasion by transwell assay. The numbers of migration and invasion cells were quantified. Wound-healing assay was performed with (**C**) SMMC-7721, (**D**) Hep-G2, (**G**) MHCC-LM3, and (**H**) Sk-hep-1 cells. Cell migration was quantified as percentage of wound-healed area. Data are means ± s.d. ^*^P < 0.05. NC, negative control siRNA; siRNA1& siRNA 2, siRNAs directed at CFIm25.

Cell invasion and migration are critical during the multistep process of tumor metastasis. To assess whether CFIm25 is an important factor in cell invasion and migration, we used RNA interference (RNAi) to suppress CFIm25 expression in SMMC-7721 and Hep-G2 cells, and we used full-length plasmid (pcDNA3.1- CFIm25) to stably express CFIm25 in Sk-hep-1 and MHCC-LM3 cells. Sk-hep-1 and MHCC-LM3 cells stably expressing CFIm25 inhibited the invasion and migration ability compared with control cells (Figure 3E, 3F), and loss of CFIm25function in SMMC-7721 and Hep-G2 cells promoted invasion and migration (Figure 3A, 3B). To confirm this result, we utilized a wound-healing assay to evaluate the effect of CFIm25 on cell migration. Consistent with previous observations, the overexpression of CFIm25 inhibited (Figure 3G, 3H) whereas knockdown CFIm25 enhanced the mobility of HCC cells (Figure 3C, 3D). These results prove that expression of CFIm25 correlates with the invasion and migration ability in HCC cells.

### CFIm25 is a factor in the EMT process

**Figure 4:**
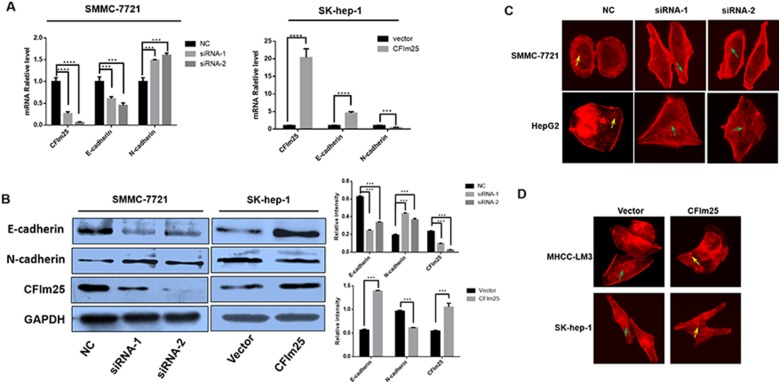
CFIm25 is active in the EMT process (**A**) Real-time RT-PCR and (**B**) Western blot show that CFIm25 suppression decreases the expression of E-cadherin and increases N-cadherin in SMMC-7721, whereas overexpression of CFIm25 in Sk-hep-1 increases the expression of E-cadherin and decreases N-cadherin. (**C**) and (**D**) CFIm25 inhibition impairs epithelial phenotype of HCC cells. F-actin cytoskeletal arrangement was examined by fluorescence microscopy in (C) SMMC-7721, Hep-G2, and (D) MHCC-LM3, Sk-hep-1, green arrows: cortical F-actin organized as curvilinear network, yellow arrows: punctate F-actin. Data are means ± s.d. ^*^P < 0.05. NC, negative control siRNA; siRNA1& siRNA 2, siRNAs directed at CFIm25.

EMT is an important mechanism of tumor invasion and metastasis. We further explored whether the CFIm25 blockade of tumor cell invasion and metastasis is through the EMT process. We found that overexpression of CFIm25 increases the expression of E-cadherin, a marker protein of EMT. At the same time, the expression of N-cadherin is decreased. With inactive CFIm25, the result is the opposite (Figure 4A, 4B). To prove that CFIm25 can bind to the cytoskeletons of hepatocarcinoma cells, we used immunofluorescent microscopy to examine the effect of CFIm25 on the F-actin cytoskeletal arrangement. We suppressed CFIm25 expression in SMMC-7721 and Hep-G2 cells. When CFIm25 is inhibited, F-actin fibers in the culture are densely arranged just inside the cell periphery, where they form a circular bundle; slim central fibers are also visible. This organization is characteristic of cells with apical-basolateral polarity, such as epithelial cells. Excessive expression of CFIm25 in Sk-hep-1 and MHCC-LM3 cells yields the opposite result (Figure 4C, 4D).

### CFIm25 inhibits HCC cell migration and invasion by blocking the p38 and JNK/c-Jun signal pathways

**Figure 5:**
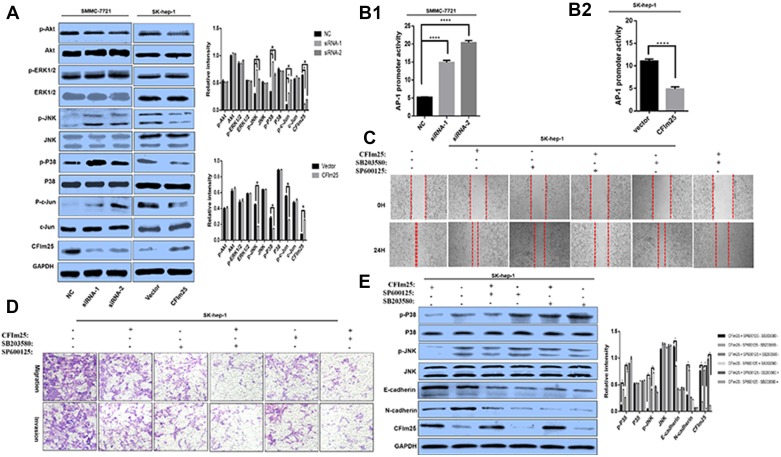
CFIm25 inhibits HCC cell migration and invasion through suppression of the p38 and JNK/c-Jun signal pathways (**A**) Protein levels of CFIm25, phosphorylated and total Akt, ERK1/2, p38, JNK, and c-Jun were analyzed by Western blot. (**B1**) CFIm25 inhibition increases the transcriptional activity of AP-1 in SMMC-7721, as determined by luciferase reporter assay. (**B2**) Overexpression of CFIm25 decreased the transcriptional activity of AP-1 in Sk-hep-1. Migration and invasion of the HCC cells were disrupted after addition of p38 inhibitor SB203580 (10 μM) and JNK inhibitor SP600125 (10 μM) by (**C**) wound-healing assay and (**D**) transwell assay in Sk-hep-1. (**E**) Protein levels of CFIm25, E-cadherin, N-cadherin, phosphorylated and total p38, and JNK were analyzed with SB203580 (10 μM) and SP600125(10 μM) pretreatment in Sk-hep-1. Data are means ± s.d. ^*^P < 0.05. NC, negative control siRNA; siRNA1& siRNA 2, siRNAs directed at CFIm25.

To address the molecular mechanism of CFIm25 defense against HCC cell invasion and metastasis, we investigated the MAPKs and Akt signal pathways, which are two main pathways for tumor proliferation and metastasis [17–20]. Inhibition of CFIm25 led to increased levels of phosphorylated JNK, p38, and c-Jun in HCC cells, but no differences were seen in phosphorylated Akt and ERK1/2 (Figure 5A). Activation of c-Jun by CFIm25 knockdown was further confirmed by the activation of its direct downstream effector, c-Jun-driven activating protein-1 (AP-1), as evidenced by luciferase reporter assay (Figure 5B).

To clarify stimulation of CFIm25-induced E-cadherin expression by the p38 pathway, the JNK/c-Jun pathway, or both, SB203580 and SP600125 were used inactivate p38 and JNK/c-Jun, respectively. Pretreatment with the JNK inhibitor (SP600125) and the p38 inhibitor (SB203580) reduced CFIm25-induced E-cadherin expression (Figure 5E). Wound-healing and transwell assay results from woverexpression of CFIm25 in Sk-hep-1 cells were confirmed through downregulation of p38 and JNK/c-Jun phosphorylation (Figure 5C, 5D). Our results indicate that CFIm25 upregulate E-cadherin expression in HCC cells, which are dependent on the p38 and JNK/c-Jun signal pathways.

### Overexpression of CFIm25 inhibits intrahepatic metastasis and lung metastasis *in vivo*

**Figure 6:**
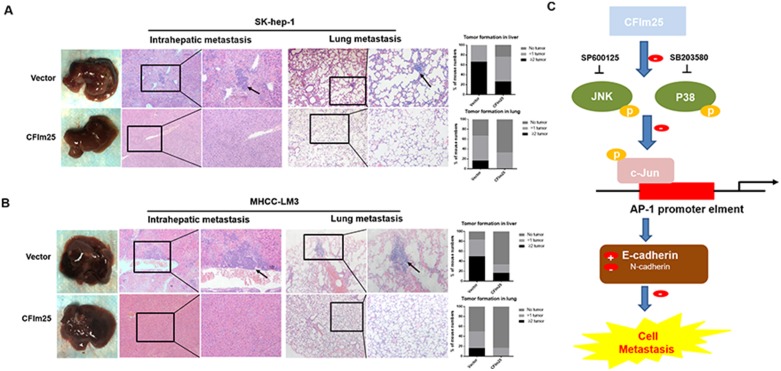
Overexpression of CFIm25 inhibits intrahepatic metastasis and lung metastasis *in vivo* Representative H&E staining of livers and lungs after injected (**A**) Sk-hep-1 and (**B**) MHCC-LM3 cells that had high metastatic potential stably expressing CFIm25. Black arrow indicates the intrahepatic or lung metastatic tumors. (**C**) Mechanistic scheme of CFIm25 in HCC metastasis.

Having observed that CFIm25 inhibited HCC cells migration *in vitro*, we confirmed the anti-metastasis function of CFIm25 *in vivo*. We injected nude mice with Sk-hep-1 and MHCC-LM3 cells that had high metastatic potential stably expressing CFIm25. The mice injected with Sk-hep-1-CFIm25 and MHCC-LM3- CFIm25 cells showed less intrahepatic and lung metastasis, compared with the control mice (Figure 6A, 6B). These results imply that CFIm25 suppresses HCC metastasis.

## DISCUSSION

HCC is prone to early metastasis, which is one of the main causes of cancer-related death. Investigations have demonstrated that normal embryogenesis and neoplasia share many basic processes and molecular pathways. Tumorigenesis may result from the loss of control by normal developmental genes [21]. APA, a post-transcriptional modification implicated in many diseases (hematological disorders [22–24], infection and immunological conditions [25–27], neurological diseases [28–30], and endocrine diseases [31, 32]), including cancer [33, 34]. The closely coupled cleavage and polyadenylation reactions are key steps in the processing of most primary pre-mRNA transcripts into mature mRNAs [35]. Understanding of this process has improved since the initial identification of a common AAUAAA polyadenylation signal (PAS) approximately 20 nt upstream from the cleavage site [36]. The global shortening of messenger RNAs through APA that occurs during enhanced cellular proliferation is a poorly understood mechanism of gene expression [6, 37]. CFIm25 is a key factor in APA [38, 39]. Studies show that CFIm25 promotes the anti-tumor effect in glioblastoma [13]. These findings identify activity of CFIm25 in suppressing APA and reveal a connection between CFIm25 and glioblastoma tumorigenicity [13]. Ospina-Villa et al. [40] observed that Leu135 and Tyr236 residues accelerate RNA binding activity of CFIm25 in *Entamoeba histolytica*. Fukumitsu et al. [41] showed that knockdown of CFIm25 promotes neurite outgrowth in developing neurons by coordinating activity upstream of NGF-induced RhoA inactivation [41]. However, no studies show that CFIm25 is associated with HCC.

In this study, we found a differential expression of CFIm25 between HCC and non-cancerous tissues We also found that CFIm25 expression is associated with TMN staging and progression of human HCC. This result suggests that CFIm25 might be active in HCC metastasis. Little evidence suggests that APA is associated with tumor metastasis. A study shows that the RhoA guanine exchange factor NET1, which is a factor in the metastasis of gastric cancer, isoforming with short 3′ UTR, promotes cellular migration and invasion *in vitro* [42], but CFIm25 and metastases in HCC has not been studied.

We tried to determine the relation between CFIm25 and HCC metastasis. We identified CFIm25 expressions in HCC cell lines with different metastatic potentials and found that CFIm25 expression is contrary to the invasiveness of HCC cells. This finding implies that CFIm25 might have anti-metastasis properties. We utilized transwell and wound-healing assays to detect the relation between CFIm25 and metastasis in four HCC cells lines (Sk-hep-1, MHCC-LM3, Hep-G2, and SMMC-7721) *in vitro*. The results were consistent with our expectations.

Based on the previous results, we investigated CFIm25disruption of the metastasis process. EMT is a universal form of tumor metastasis and E-cadherin is an important factor in the EMT process, as is a component in the formation of cell-cell adherens junctions in epithelial tissues [43]. Studies show that suppression of E-cadherin results in mesenchymal morphology and increased cell migration and invasion, as well as metastasis [44]. The experimental and physiological observations regarding the tumor suppressor role of E-cadherin are often interpreted in the context of EMT [45]. The EMT is a reversible and dynamic process that allows the transition between the epithelial and the mesenchymal phenotype during tissue development and repair. It results in the conversion of tightly connected epithelial cells into more loosely adherent, fibroblast-like cells [46]. Research has demonstrated that re-introduction of E-cadherin into cell lines in which it is depleted promotes reversion of poorly differentiated carcinoma phenotypes (i.e., fibroblastic, highly invasive, poorly cell-cell adherent) to well-differentiated, minimally invasive epithelioid phenotypes with well-developed cell-cell junctions [47]. Depletion of E-cadherin activates oncogenic signaling pathways, including mitogen activated kinase (MAPK), rat sarcoma viral oncogene (Ras), and ras-related C3 botulinum toxin substrate (Rac1), and disrupts the Hippo signaling pathway, which leads to cell migration and invasion [44]. Our study found that the expression of E-cadherin was low/high after high/low expression of CFIm25, and the expression of N-cadherin was opposite to that of E-cadherin. This finding suggests that CFIm25 might prevent the metastasis of HCC by suppressing the EMT process.

With further research, we demonstrated that the anti-metastatic effect of CFIm25 in SMMC7721 cells was through blocking the p38 and JNK/c-Jun pathways, but no total ERK or Akt influence. JNK and p38 mitogen-activated protein kinases (MAPKs) have important functions in the signaling mechanisms that orchestrate cellular responses to many types of stresses, but they also control the proliferation, differentiation, survival and migration of specific cell types [48]. Transcription factor AP-1 is associated with JNK, which consists of c-Fos and c-Jun family members [49]. The oncogenic functions of JNK are mostly based on their ability to phosphorylate Jun and to activate AP-1, whereas their tumor-suppressive functions are probably related to their pro-apoptotic activity. Studies show that cross communication between the JNK and p38 MAPK pathways is as a controlling mechanism in many cellular responses [48]. We used siRNA, which diminishes the function of CFIm25, to activate the p38 and JNK/c-Jun signaling cascade, subsequently activating the AP-1 transcription that stimulates the downstream genes involved in promoting HCC cell metastasis.

Previous studies show that CFIm25 inhibits the invasion and metastasis of hepatocellular carcinoma by blocking the JNK/c-Jun signaling pathway and influencing the activity of AP-1 *in vitro*, and we verified this result *in vivo*. The results of *in vivo* experiments compared with our *in vitro* experiments show the same p38 and JNK/c-Jun signaling cascades.

CFIm25 is crucial for preventing invasion and metastasis of HCC. HCC upregulates AP-1 by stimulating the p38 and JNK/c-Jun signaling pathways. Thus, enhancing CFIm25 and repressing the direct downstream effector of p38 and JNK/c-Jun signaling pathways have a potential clinical application for HCC treatment.

## MATERIALS AND METHODS

### Cell lines and HCC specimens

All HCC specimens and matched nontumor tissues were collected during surgical resection at Tongji Hospital. Tumor histopathology was confirmed by analysis of hematoxylin and eosin (H&E) stained tissue sections by a qualified neuropathologist. The present study was conducted according to the guidelines of the Ethics Committee of the Tongji Hospital and in accordance with the ethical standards of the World Medical Association Declaration of Helsinki. Human HCC cell lines Huh7, SMMC7721, HepG2, MHCC LM3, and SK-Hep-1were cultured in DMEM medium supplemented with 10% fetal calf serum (Invitrogen Gibco, Carlsbad, CA, USA) and incubated in a 5% CO_2_ incubator at 37°C.

### RNA extraction and real-time RT-PCR

Total RNA was extracted by use of TRIzol Reagent (Invitrogen, Carlsbad, CA, USA). Reverse-transcribed complementary DNA was synthesized by use of a PrimeScript RT Reagent Kit (TaKaRa, Otsu, Japan). Real-time polymerase chain reaction was performed by use of SYBR Premix ExTaq (TaKaRa, Otsu, Japan) on an ABI StepOne Real-Time PCR System (Applied Biosystem, Carlsbad, CA, USA). The value of 2^–ΔΔ^Ct was used to determine fold difference between samples. The primers used for PCR sequences were as follows: GAPDH: 5′-TCATTGACCTCAACTACATGGTTT-3′ (sense) and 5′-GAAGATGGTGATGGGATTTC-3′ (antisense); CFIm25:5′- GGTCACTCAGTTCGGCAACAA-3′ (sense) and 5′- CTCATGCGCTGAAATCTGGC-3′(antisense)

### RNA interference and establishment of stable expressing cells

For RNA interference, transfection was performed by use of Lipofectamine 2000 (Life Technologies) following the standard method. The siRNA sequences (Ribo Company, Guangzhou, China) used are the following: CFIm25-siRNA1: forward 5′ GCUCUGUUGCAGCCAGAUU dTdT 3′ and reverse 3′ dTdT CGAGACAACGUCGGUCUAA 5′; CFIm25-siRNA2: forward 5′ GCACCAUUGUUUGAAUUGU dTdT 3′ and reverse 3′ dTdT CGUGGUAACAAACUUAACA 5′. For establishment of stable expressing cells, plasmids (Genechem Company, China) were transfected into cells with Lipofectamine 2000 according to the manufacturer’s instructions. We achieved the stable overexpression transfectant by adding G418 (Sigma Aldrich) for 4 weeks.

### *In vitro* migration and invasion assay

Migration assays were conducted by use of 24-well plate containing inserts (8-μm pores; Costar, USA). Invasion assays were performed similarly by use of matrigel-coated inserts (Costar, USA). The lower chamber was filled with 600-μL medium containing 12% serum, and the top chamber contained 2 × 10^4^ cells suspended in 200-μL medium without serum. After 24 hours of incubation, cells invading into the bottom side of the inserts were fixed, stained, photographed, and quantified by counting them in five random fields. Wound-healing assays were conducted on indicated cells. Cells were plated to confluence in 6-well plates. After the cells had adhered to the plates, streaks were created in the monolayer with a pipette tip. Progression of migration was observed and photographed at 0 and 24 hours after wound.

### *In vivo* metastasis assays

For mouse tail vein injection, 1 × 10^5^ cells in 100 μL of phosphate buffered saline (PBS) were injected into the tail veins of nude mice. The mice were sacrificed on day 30. Liver and lung tissues were resected and fixed with 4% paraformaldehyde, and then strained with H&E.

### Western blot analysis

Western blot analysis were performed as previously described [50].

### Immunohistochemistry staining

Paraffin-embedded tissues were cut into four μm-thick consecutive sections and were then dewaxed in xylene and rehydrated in graded ethanol solutions. Tissue sample slides were deparaffinized with dimethylbenzene, followed by gradient alcohol dehydration, and incubation with 3% hydrogen peroxide to block endogenous peroxidase, and then to block non-specific binding sites. Primary anti-CFIm25 antibody (1:400, ABcam, USA) was incubated overnight at 4° C. The secondary antibody was added for 1 hour at room temperature, and then developed by peroxidase conjugated streptavidin and diaminobenzidine, and counterstained with hematoxylin.

The intensity of staining was scored on a four-point scale as negative (0), weak (1), moderate (2), and strong (3). The extent of the staining, defined as the percentage of positively stained tissue area in relation to the entire tissue area, was scored on a scale of 0 to 4: 0 (0%), 1 (1%–25%), 2 (26%–50%), 3 (51%–75%), and 4 (76%–100%) [51]. Finally, the immunohistochemistry score was equal to staining intensity multiplied by staining extent [52]. The result greater than or equal to 5 was defined as high expression, and less than 5 was defined as low expression.

### Luciferase reporter experiments

AP-1 reporter activity was detected by a Secrete-PairTM Dual Luminescence Assay Kit (GeneCopoeia) according to the manufacturer’s instructions.

### Statistical analyses

All experiments were performed in triplicate unless specified. The data are presented as mean ± SD. Statistical analyses were performed by Student’s *t*-test. The Pearson chi-square was used to analyze the correlation between CFIm25expression and pathogenic conditions of chronic liver disease in human samples. Statistical analyses were performed by Prism 5.0 (GraphPad Software, La Jolla, CA, USA). *A* value of *P* < 0.05 was considered statistically significant.
